# Causal association between air pollution and autoimmune diseases: a two-sample Mendelian randomization study

**DOI:** 10.3389/fpubh.2024.1333811

**Published:** 2024-03-28

**Authors:** Haiping Hu, Xinxin Yang, Qingquan Chen, Xinfeng Huang, Xiangyu Cao, Xiaoyang Zhang, Youqiong Xu

**Affiliations:** ^1^The Affiliated Fuzhou Center for Disease Control and Prevention of Fujian Medical University, Fuzhou, China; ^2^School of Public Health, Fujian Medical University, Fuzhou, China

**Keywords:** air pollution, autoimmune diseases, Mendelian randomization, particulate matter, nitrogen oxides

## Abstract

**Background:**

In recent years, an increasing number of observational studies have reported the impact of air pollution on autoimmune diseases (ADs). However, no Mendelian randomization (MR) studies have been conducted to investigate the causal relationships. To enhance our understanding of causality, we examined the causal relationships between particulate matter (PM) and nitrogen oxides (NO_x_) and ADs.

**Methods:**

We utilized genome-wide association study (GWAS) data on PM and NO_x_ from the UK Biobank in European and East Asian populations. We also extracted integrated GWAS data from the Finnish consortium and the Japanese Biobank for two-sample MR analysis. We employed inverse variance weighted (IVW) analysis to assess the causal relationship between PM and NO_x_ exposure and ADs. Additionally, we conducted supplementary analyses using four methods, including IVW (fixed effects), weighted median, weighted mode, and simple mode, to further investigate this relationship.

**Results:**

In the European population, the results of MR analysis suggested a statistically significant association between PM_2.5_ and psoriasis only (OR = 3.86; 95% *CI*: 1.89–7.88; *P*_IVW_ < 0.00625), while a potential association exists between PM_2.5–10_ and vitiligo (OR = 7.42; 95% *CI*: 1.02–53.94; *P*_IVW_ < 0.05), as well as between PM_2.5_ and systemic lupus erythematosus (OR = 68.17; 95% *CI*: 2.17–2.1e+03; *P*_IVW_ < 0.05). In East Asian populations, no causal relationship was found between air pollutants and the risk of systemic lupus erythematosus and rheumatoid arthritis (*P*_IVW_ > 0.025). There was no pleiotropy in the results.

**Conclusion:**

Our results suggest a causal association between PM_2.5_ and psoriasis in European populations. With the help of air pollution prevention and control, the harmful progression of psoriasis may be slowed.

## Background

Autoimmune diseases (ADs), as products of the intertwined effects of innate genetic factors and environmental triggers, have attracted extensive attention worldwide. These diseases cause multiple immune system disorders (1), affect nearly 5% of the global population, and their prevalence and incidence are on the rise (2). It has a broad spectrum of diseases, including inflammatory bowel disease, rheumatoid arthritis, systemic lupus erythematosus, and psoriasis, among others. They are not congenital diseases and can develop at any age, so it is particularly critical to explore their predisposing factors. However, the pathogenesis of ADs has not yet been fully clarified, and multiple risk factors such as genetic (3), immune (4), and environmental (2) are thought to be important in increasing their risk.

As people’s health awareness increases, the threat of environmental factors to health is gradually being emphasized. Studies have shown that environmental factors account for 40–70% of the development of ADs (5, 6). In retrospect, air pollution, as one of the major risk factors for the environment, has been temporally and strongly associated with the global increase in the incidence of type 2 diabetes mellitus (7), ADs (8) and cardiovascular diseases (9). In recent years, an increasing number of studies have linked ambient air pollution to the occurrence and development of ADs, suggesting that pollutants such as PM_2.5_, PM_2.5–10_, PM_10_, and nitrogen oxides (NO_x_) may increase the risk of diseases such as systemic lupus erythematosus (10), rheumatoid arthritis (11), inflammatory bowel disease (12), and psoriasis (13). These findings have been validated not only in European populations but also in East Asian populations (14, 15).

However, on the other hand, some studies have shown no association between air pollutants and the risk of developing rheumatoid arthritis (11, 16), inflammatory bowel disease (17) or multiple sclerosis disease (18, 19). This may be related to limitations of observational studies, such as insufficient adjustment for confounders, limited follow-up time, or small sample size. Therefore, many challenges remain to establish a clear causal relationship between air pollution and ADs. To overcome these limitations, we plan to use Mendelian randomization (MR) analysis as a tool to assess the causal relationship between PM_2.5_, PM_2.5–10_, PM_10_ and NO_x_ with systemic lupus erythematosus, rheumatoid arthritis, inflammatory bowel disease, vitiligo, multiple sclerosis disease, myasthenia gravis, coeliac disease and psoriasis. This approach is less susceptible to confounding bias and risk of reverse causation, and is expected to provide us with more accurate and reliable evidence to reveal the potential link between air pollution and ADs.

## Methods

### Study design

**Figure 1:**
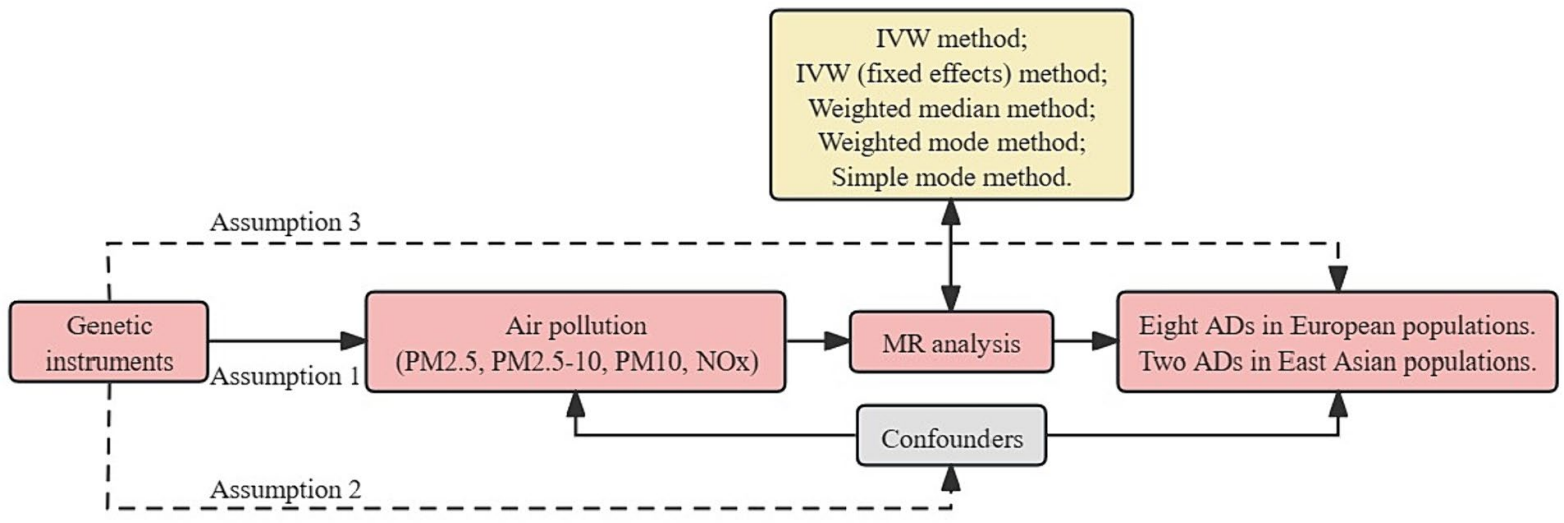
Overview flowchart of assumptions and schematic design. SNPs associated with air pollution were used as genetic instruments to study the causal effect of air pollution on frailty. Lines with arrows indicate that genetic instruments (SNPs) are associated with exposure and can only influence the outcome through exposure. Dashed lines indicate that the genetic tools (SNPs) are not associated with confounders between the results. ADs, autoimmune diseases; IVW, inverse variance weighted; MR, Mendelian randomization; NOx, nitrogen oxides; PM, particulate matter; SNPs, single nucleotide polymorphisms.

MR analysis is based on three essential assumptions. The first assumption is that the genetic variants proposed as instrumental variables should have a robust association with the exposure. The second assumption states that the chosen genetic variants should not be associated with any confounding factors. The third assumption is that the selected genetic variants should only affect the risk of the outcome through risk factors (Figure 1). This MR investigation is based on publicly available GWAS, and all included studies have received approval from the respective institutional review boards and ethics committees.

### Data source

The dataset containing information on air pollutants was acquired from the UK Biobank’s Metadata of Environmental Exposures. To estimate air pollutants levels for the year 2010 at each address, a Land Use Regression (LUR) model developed as part of the European Study of Cohorts for Air Pollution Effects (ESCAPE) was utilized. The ESCAPE project received funding under the EU 7th Framework Program. The LUR model is based on monitoring conducted between January 26, 2010, and January 18, 2011, and the resulting air pollution estimates are representative of the year 2010.

Genetic associations for the eight ADs were obtained from the latest summary-level genetic data for European populations from the FinnGen study. In FinnGen, genome-wide association analysis was adjusted for gender, age, genetic ancestry, and genotyping batch. Genetic associations for two ADs in East Asian populations were also acquired from the Japan Biobank research and Wang YF (Supplementary Table 1).

### Screening of genetic instruments

Genetic instrumental variables for environmental pollutants in European and East Asian populations were extracted from the latest GWAS data. The Medical Research Council Integrative Epidemiology Unit (MRC-IEU) conducted meta-analysis on GWAS data for environmental pollutants from the UK Biobank (Supplementary Table 1). A threshold of *p* < 5 × 10^−8^ was used to identify Single Nucleotide Polymorphisms (SNPs) significantly associated with PM_2.5_, PM_10_, and NO_x_ exposure in European populations. Due to an insufficient number of SNPs, a threshold of *p* < 5 × 10^−6^ was used for PM_2.5–10_ exposure in European populations and all exposures in East Asian populations. SNPs were defined as not in linkage disequilibrium if *r^2^* > 0.01 and clump distance >10,000 kb. Weak instrumental variable bias was assessed using *F*-statistics, ensuring that all SNPs had *F*-statistics greater than 10, thus confirming a strong correlation between instrumental variables and all exposures (Supplementary Table 2).

### Statistical analysis

We evaluated the causal relationship between air pollutants and ADs using five MR methods. The primary method for MR analysis was the inverse variance weighted (IVW) method (20). Mendelian randomization Multi-Phenotype Residual Sum and Outlier (MR-PRESSO) was used to detect outliers in IVW linear regression and correct MR estimates after their removal. Supplementary methods included IVW (fixed effects), weighted median, weighted mode, and simple mode (Figure 1). Also, when the IVW method is statistically significant and the other methods are not, the OR value of the other methods must be in the same direction as the IVW, otherwise it is considered not statistically significant (21).

Sensitivity analyses were performed using various methods to confirm the robustness and validity of the results. Firstly, to assess heterogeneity between SNP estimates, Cochran’s *Q*-statistic was utilized. Secondly, to assess horizontal pleiotropy among SNP estimates, MR-Egger regression (22) and MR-PRESSO (20) global tests were employed for outlier detection. After removing outliers, IVW estimates without pleiotropy had a statistical threshold of *p* > 0.05. Finally, we also assessed bias due to individual SNP influence on outcomes using a leave-one-out analysis.

Bonferroni-corrected *p*-values included *p* = 0.05/8 = 0.00625 for adjusting multiple tests in European MR and *p* = 0.05/2 = 0.025 for East Asian MR. All statistical tests were two-sided, and R software version 4.3.0, along with the TwoSampleMR and MR-PRESSO packages, were used for analysis.

## Results

**Figure 2:**
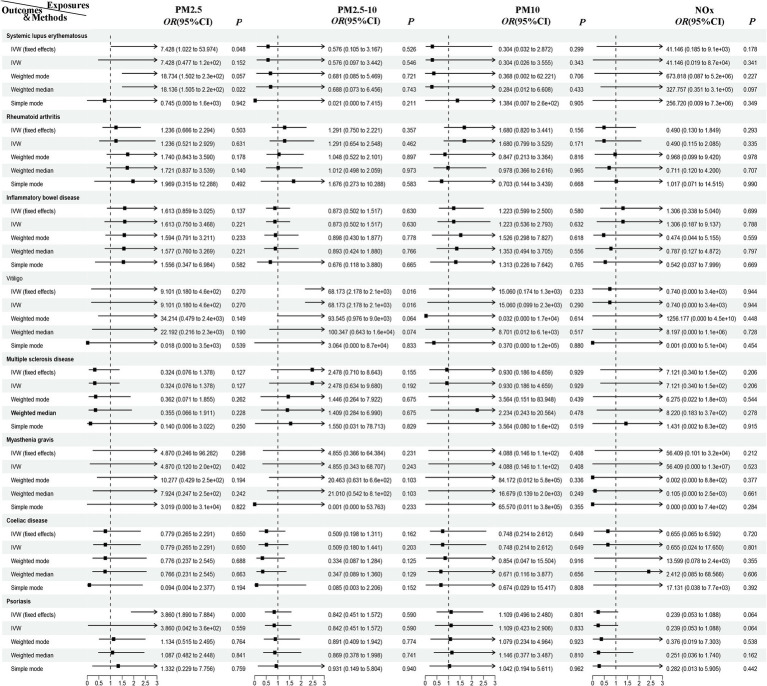
MR analysis of air pollution to ADs in European population. PM, particulate matter; NO_x_, nitrogen oxides; OR, odds radio; CI, confidence interval; IVW, inverse variance weighted; MR, Mendelian randomization; ADs, autoimmune diseases.

**Figure 3:**
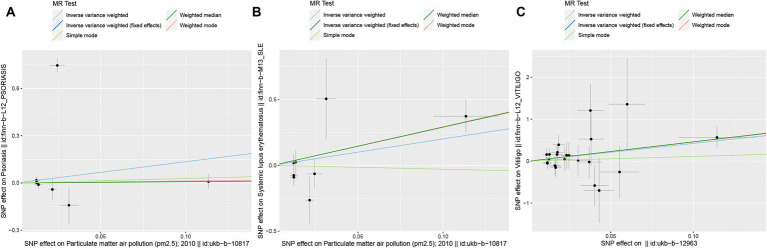
Scatter plots of SNPs associated with air pollution and ADs. Each black point representing each SNP on the exposure (horizontal-axis) and on the outcome (vertical-axis) is plotted with error bars corresponding to each standard error. The MR regression slopes of the lines represent the causal estimates using five approaches (IVW, IVW (fixed effects), simple mode, weighted median, and weighted mode). **(A)** PM_2.5_. **(B)** PM_2.5_. **(C)** PM_2.5–10_. MR, Mendelian randomization; SNP(s), single nucleotide polymorphism(s); ADs, autoimmune diseases; IVW, inverse variance weighted; PM, particulate matter.

In the European population, there is a correlation between PM_2.5_ levels and psoriasis (*P*_ivw_ < 0.00625), while a potential association exists between PM_2.5–10_ and vitiligo, as well as between PM_2.5_ and systemic lupus erythematosus (*P*_ivw_ < 0.05). Our results also show no support for the causal hypothesis that the remaining diseases are related to air pollution (*P*_ivvw_ > 0.00625) (Figures 2, 3). Furthermore, there is no evidence of significant horizontal pleiotropy (*P*_pleiotropy_ > 0.05) (Supplementary Table 3). When heterogeneity is present, MR-PRESSO was used to remove heterogeneous SNPs (*P*_Cochrane’s Q_ > 0.05 and *P*_MR-PRESSO_ > 0.05) (Supplementary Tables 3, 4). Leave-one-out analysis (Supplementary Table 5) indicates that removing each SNP one by one had little impact on the results, suggesting that no single SNP significantly influenced the overall causal effect estimate.

**Figure 4:**
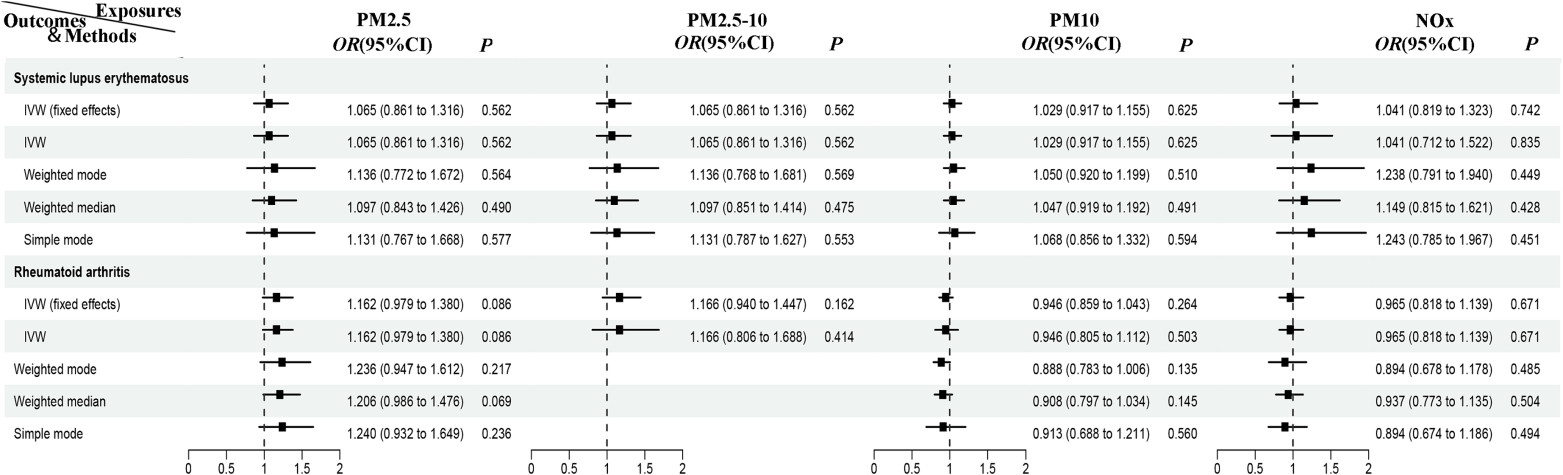
MR analysis of air pollution to systemic lupus erythematosus and rheumatoid arthritis in East Asian population. PM, particulate matter; NO_x_, nitrogen oxides; OR, odds radio; CI, confidence interval; IVW, inverse variance weighted; MR, Mendelian randomization.

Due to the lack of GWAS data for other ADs, MR analysis was only conducted for environmental pollutants and systemic lupus erythematosus and rheumatoid arthritis in the East Asian population, further enhancing the credibility of the results mentioned above. After removing the linkage disequilibrium IVs, 8, 23, 22, and 8 SNPs were identified for PM_2.5_, PM_2.5–10_, PM_10_, and NO_x_, respectively. The results show that in the East Asian population, there is no evidence of a non-causal relationship between air pollutants and the risk of systemic lupus erythematosus and rheumatoid arthritis (*P*_ivw_ > 0.025). Using the IVW model, IVW fixed effects model, weighted model, weighted median model, and simple model methods, we found no evidence of a causal relationship between air pollutants and the two ADs (*P*_ivw_ > 0.025) (Figure 4). However, due to the lack of sufficient instrumental variables for PM_2.5–10_ and rheumatoid arthritis MR, we only provided two analytical methods. Furthermore, there is no evidence of significant horizontal pleiotropy (*P*_pleiotropy_ > 0.05) and heterogeneity (*P*_Cochrane’s Q_ > 0.05 and *P*_MR-PRESSO_ > 0.05) (Supplementary Table 3). Leave-one-out analysis (Supplementary Table 6) indicates that removing each SNP one by one had little impact on the results, suggesting that no single SNP significantly influenced the overall causal effect estimate.

## Discussion

We conducted a comprehensive MR investigation of the associations between PM_2.5_, PM_2.5–10_, PM_10_, and NO_x_ exposure and eight ADs in the European population. We further validated these associations with two ADs in the East Asian population. Multiple sensitivity analyses and MR analyses in two populations ensured the reliability of the results. We found a correlation between PM_2.5_ levels and psoriasis in individual diseases in European populations (*P*_ivw_ < 0.00625); and potential associations between PM_2.5–10_ and vitiligo as well as between PM_2.5_ and systemic lupus erythematosus (*P*_ivw_ < 0.05). However, overall, it appeared that PM_2.5_, PM_2.5–10_, PM_10_ and NO_x_ were not associated with an increased risk of the eight ADs (except PM_2.5_ and psoriasis).

In a number of diseases in European populations, our findings are similar to those of previous studies (13, 23, 24). For systemic lupus erythematosus, vitiligo, and psoriasis, the biological relationship between the effects of air pollution on these diseases has not been established. The composition of PM, a major constituent of air pollutants, is more complex and varies from region to region. Previous studies (25, 26) have suggested that dysbiosis of the intestinal microflora is one of the pathogenic mechanisms of systemic lupus erythematosus. Since bacteria are also a constituent of PM, we hypothesize that airborne bacterial particles may also be involved in the immune-inflammatory response and predispose to the development of systemic lupus erythematosus. Previous studies (27, 28) have shown that oxidative stress damage to melanocytes plays an important role in vitiligo. Exposure to PM inhibits the secretion of stem cell factor (SCF) and basic fibroblast growth factor (bFGF) in keratinocytes, causing oxidative stress damage and disruption of melanocyte melanin metabolism (24). Therefore, PM may also be a risk factor for vitiligo. It has been claimed that PM treatment of keratinocytes increases cellular reactive oxygen species (ROS) production, leading to the activation of T-helper 1 (Th1) and Th17 cells (29). Specifically, PM activates aryl hydrocarbon receptors (important sensors of environmental chemicals) and further induces the production of ROS, leading to the inflammation associated with psoriasis (30, 31).

The current MR studies confirm the findings of previous epidemiological research (32, 33). However, our study results contradict some prior observational studies (10, 12, 27, 34–37). These contradictions may arise from unmeasured confounding factors, and the relatively small sample size in these studies may contribute to these discrepancies. Some studies (2) suggest that the lungs might be the initial site where PM triggers ADs. The mechanisms driving lung cancer due to fine PM are not primarily through increased genetic mutations but rather rely on altering the immune system, creating an inflammatory microenvironment, attracting macrophages to the lungs, and stimulating the release of IL-1β. Another study proposes that the mechanisms linking exposure to air pollutants with ADs primarily involve oxidative stress leading to systemic inflammation and immune imbalance. This includes the regulation of dendritic cells, regulatory T cells, and the function and phenotype of T cells, ultimately leading to the development of ADs (38). While these mechanisms seem plausible in theory, they have yet to be definitively validated through randomized controlled experiments. Furthermore, the existing observational research lacks sufficient compelling evidence, and its results exhibit variations.

Epidemiological and clinical research has provided some evidence suggesting that PM and NO_x_ may not directly trigger ADs but could potentially prolong the duration of diseases, worsen clinical symptoms, and lead to disease relapse and other adverse effects. Therefore, implementing policies to reduce exposure to environmental pollutants, such as using filters in air conditioning systems or wearing masks in traffic, remains necessary. Some studies have indicated that PM and NO_x_ exposure may prolong the course of systemic lupus erythematosus (39), exacerbate symptoms (40–42), and lead to complications (42). Another study has suggested that air pollution has pro-inflammatory effects on multiple sclerosis disease (43) and increases the risk of multiple sclerosis disease relapse (44, 45). Furthermore, there is also research indicating that exposure to PM and NO_x_ may be associated with an increased risk of cancer (46), such as lung cancer (47). Additionally, the potential impact of other air pollutants (48) on ADs, including but not limited to ozone, kitchen fumes, nicotine, aldehydes, methane, and chlorofluorocarbons, should not be overlooked. Therefore, these research findings suggest that environmental pollutants may have varying degrees of impact on ADs and health issues like cancer, warranting further investigation into their mechanisms and the implementation of necessary measures to reduce pollutant exposure for public health maintenance.

Our study has several limitations that should be considered. Firstly, there is limited genetic data available for ADs in the East Asian population. While our MR analysis is based on a cross-ethnicity two-sample MR design, it only includes two diseases, namely rheumatoid arthritis and systemic lupus erythematosus. Future research should encompass a broader range of ADs in East Asian populations to validate the relationship between air pollutants and other ADs. Secondly, the exposure variance explained by the SNPs used as instruments for exposure is limited. In our study, the significance level for the SNPs associated with the four exposures in the East Asian population was 5e × 10^−6^. This may necessitate larger sample sizes to further validate our study’s conclusions, and future research efforts should aim to address these issues for a more comprehensive understanding of the relationship between air pollution and ADs, especially in East Asian populations.

## Conclusion

In this MR study involving four pollutants and eight ADs in European populations and four pollutants and two ADs in East Asian populations, we found significant associations between PM_2.5_ and psoriasis as well as suggestive associations between PM_2.5_ and vitiligo, and PM_2.5–10_ and systemic lupus erythematosus in the European population only, and our study did not support the remaining associations of the causal hypothesis. Therefore, the next step needs to be taken with the help of air pollution control, which can slow down the harmful progression of psoriasis.

## Data availability statement

The original contributions presented in the study are included in the article/Supplementary material, further inquiries can be directed to the corresponding authors.

## Author contributions

HH: Data curation, Formal analysis, Validation, Writing – review & editing. XY: –. QC: Conceptualization, Methodology, Validation, Writing – review & editing. XH: Formal analysis, Supervision, Writing – original draft. XC: Conceptualization, Project administration, Validation, Writing – review & editing. XZ: Conceptualization, Methodology, Project administration, Supervision, Writing – review & editing. YX: Conceptualization, Methodology, Resources, Supervision, Writing – original draft.
