# Supplementary material for: Causal association between air pollution and autoimmune diseases: a two-sample Mendelian randomization study
Source: Front Public Health. 2024 Mar 28;12:1333811. doi: 10.3389/fpubh.2024.1333811 (PMC11007215; doi:10.3389/fpubh.2024.1333811)
Supplement: Supplementary file 1 [file Data_Sheet_1.docx]

**Supplementary Table 1.** Detailed information on used studies.

| **Exposures＆Outcomes** | **Sexs** | **Sample size (cases/controls)** | **Number of SNPs** | **Year** | **Author** | **Data Sources** |
| --- | --- | --- | --- | --- | --- | --- |
| **European** | | | | | | |
| PM2.5 | combined | 423,796 | 9,851,867 | 2018 | Ben Elsworth | UKBiobank |
| PM2.5-10 | combined | 423,796 | 9,851,867 | 2018 | Ben Elsworth | UKBiobank |
| PM10 | combined | 455,314 | 9,851,867 | 2018 | Ben Elsworth | UKBiobank |
| NOx | combined | 456,380 | 9,851,867 | 2018 | Ben Elsworth | UKBiobank |
| SLE | combined | 538/213,145 | 213,145 | 2021 | NA | [FinnGen](https://www.finngen.fi/en/access_results) |
| RA | combined | 6236/147,221 | 147,221 | 2021 | NA | FinnGen |
| CD | combined | 1,973/210,964 | 16,380,438 | 2021 | NA | FinnGen |
| MG | combined | 232/217,056 | 16,380,458 | 2021 | NA | FinnGen |
| Psoriasis | combined | 4,510/ 212,242 | 16,380,464 | 2021 | NA | FinnGen |
| MSD | combined | 1,048/217,141 | 16,380,460 | 2021 | NA | FinnGen |
| IBD | combined | 5,673/213,119 | 16,380,466 | 2021 | NA | FinnGen |
| Vitiligo | combined | 131/207,482 | 16,380,442 | 2021 | NA | FinnGen |
| **East Asian** | | | | | | |
| PM2.5 | combined | 2,505 | 8,268,350 | 2020 | Pan-UKB team | UKBiobank |
| PM2.5-10 | combined | 2,506 | 8,268,351 | 2020 | Pan-UKB team | UKBiobank |
| PM10 | combined | 2,507 | 8,268,352 | 2020 | Pan-UKB team | UKBiobank |
| NOx | combined | 2,508 | 8,268,353 | 2020 | Pan-UKB team | UKBiobank |
| SLE | combined | 4,222/8,431 | 5,691,661 | 2021 | Wang YF | 33536424 |
| RA | combined | 4,199/208,254 | 8,885,805 | 2019 | NA | BioBank Japan |

PM: Particulate matter; SLE: Systemic lupus erythematosus; RA: Rheumatoid arthritis;CD: coeliac disease; MG: Myasthenia gravis; MSD: Multiple sclerosis disease;IBD: Inflammatory bowel disease; NOx: Nitrogen Oxides.

**Supplementary Table 2.** Detailed information on genetic instruments of exposures.

| **Exposure** | **BETA** | **SE** | ***P*-value** | **SNP** | **Effect_allele** | | **Other_allele** | | **EAF** | **F-statistic** | | |
| --- | --- | --- | --- | --- | --- | --- | --- | --- | --- | --- | --- | --- |
| **European** | | | | | | | | | | | |  |
| PM2.5 | -0.012 | 0.002 | 1.40E-08 | rs6749467 | A | G | | 0.466 | | | 32.228 |  |
|  | 0.012 | 0.002 | 3.10E-08 | rs1372504 | A | G | | 0.374 | | | 30.674 |  |
|  | 0.022 | 0.003 | 6.20E-17 | rs12203592 | T | C | | 0.213 | | | 69.918 |  |
|  | 0.025 | 0.004 | 4.20E-08 | rs114708313 | T | A | | 0.066 | | | 30.076 |  |
|  | 0.031 | 0.006 | 4.20E-08 | rs77255816 | T | C | | 0.037 | | | 30.041 |  |
|  | 0.014 | 0.002 | 2.10E-08 | rs77205736 | T | C | | 0.274 | | | 31.399 |  |
|  | 0.012 | 0.002 | 8.50E-09 | rs1537371 | A | C | | 0.500 | | | 33.149 |  |
|  | 0.113 | 0.019 | 3.10E-09 | rs72642437 | T | C | | 0.004 | | | 35.119 |  |
| PM2.5-10 | 0.017 | 0.003 | 1.90E-06 | rs76170056 | A | C | | 0.111 | | | 22.736 |  |
|  | 0.016 | 0.003 | 1.70E-06 | rs71323440 | T | C | | 0.115 | | | 22.859 |  |
|  | 0.011 | 0.003 | 4.70E-06 | rs13125748 | A | C | | 0.258 | | | 20.959 |  |
|  | -0.010 | 0.002 | 3.40E-06 | rs9997134 | C | T | | 0.399 | | | 21.570 |  |
|  | -0.043 | 0.009 | 2.70E-06 | rs138141967 | T | G | | 0.015 | | | 21.997 |  |
|  | 0.038 | 0.008 | 2.30E-06 | rs116816317 | A | G | | 0.020 | | | 22.343 |  |
|  | 0.030 | 0.006 | 1.10E-06 | rs116259145 | A | C | | 0.032 | | | 23.784 |  |
|  | -0.038 | 0.008 | 7.70E-07 | rs78060907 | A | C | | 0.020 | | | 24.439 |  |
|  | -0.013 | 0.003 | 7.20E-07 | rs9497937 | A | C | | 0.232 | | | 24.556 |  |
|  | 0.037 | 0.008 | 2.80E-06 | rs17675316 | G | A | | 0.022 | | | 21.932 |  |
|  | -0.022 | 0.005 | 3.50E-06 | rs111308789 | A | T | | 0.057 | | | 21.524 |  |
|  | 0.018 | 0.004 | 6.00E-07 | rs1706918 | A | G | | 0.099 | | | 24.906 |  |
|  | -0.011 | 0.002 | 3.20E-06 | rs57048268 | C | A | | 0.311 | | | 21.705 |  |
|  | 0.060 | 0.011 | 6.50E-08 | rs118101191 | T | G | | 0.010 | | | 29.208 |  |
|  | -0.025 | 0.005 | 5.20E-07 | rs1157546 | C | T | | 0.053 | | | 25.205 |  |
|  | -0.040 | 0.009 | 2.20E-06 | rs117389221 | C | T | | 0.017 | | | 22.385 |  |
|  | -0.011 | 0.002 | 3.40E-06 | rs605027 | T | C | | 0.730 | | | 21.565 |  |
|  | -0.018 | 0.004 | 3.30E-06 | rs8006373 | A | T | | 0.091 | | | 21.659 |  |
|  | -0.023 | 0.004 | 2.50E-07 | rs11621531 | A | G | | 0.064 | | | 26.565 |  |
|  | 0.114 | 0.023 | 1.00E-06 | rs10152521 | C | T | | 0.003 | | | 23.835 |  |
|  | 0.016 | 0.003 | 1.30E-06 | rs8051340 | G | C | | 0.123 | | | 23.356 |  |
|  | -0.017 | 0.004 | 3.60E-06 | rs62079137 | C | T | | 0.107 | | | 21.490 |  |
|  | -0.012 | 0.002 | 6.90E-07 | rs12462492 | T | G | | 0.257 | | | 24.642 |  |
|  | 0.055 | 0.012 | 4.20E-06 | rs117125329 | G | C | | 0.008 | | | 21.162 |  |
| PM10 | -0.012 | 0.002 | 2.10E-08 | rs182549 | T | C | | 0.739 | | | 31.357 |  |
|  | -0.055 | 0.010 | 1.00E-08 | rs114789974 | A | C | | 0.010 | | | 32.832 |  |
|  | 0.015 | 0.002 | 5.90E-10 | rs56084453 | G | A | | 0.210 | | | 38.352 |  |
|  | -0.014 | 0.002 | 3.50E-08 | rs13084230 | T | C | | 0.200 | | | 30.395 |  |
|  | -0.013 | 0.002 | 6.60E-09 | rs6793835 | A | G | | 0.264 | | | 33.661 |  |
|  | 0.025 | 0.002 | 1.70E-25 | rs4833095 | C | T | | 0.207 | | | 108.965 |  |
|  | -0.014 | 0.002 | 1.30E-08 | rs13122455 | T | C | | 0.200 | | | 32.293 |  |
|  | -0.031 | 0.005 | 1.80E-09 | rs6867849 | T | A | | 0.040 | | | 36.198 |  |
|  | 0.040 | 0.007 | 4.40E-08 | rs142169179 | A | G | | 0.020 | | | 29.971 |  |
|  | 0.012 | 0.002 | 7.80E-09 | rs2248162 | C | T | | 0.640 | | | 33.331 |  |
|  | -0.035 | 0.006 | 1.30E-08 | rs140295641 | A | T | | 0.027 | | | 32.406 |  |
|  | -0.014 | 0.002 | 2.70E-12 | rs9640029 | T | C | | 0.478 | | | 48.898 |  |
|  | 0.016 | 0.003 | 6.40E-09 | rs61620752 | G | T | | 0.148 | | | 33.720 |  |
|  | 0.012 | 0.002 | 2.50E-08 | rs2004679 | C | T | | 0.308 | | | 31.049 |  |
|  | 0.022 | 0.004 | 6.80E-09 | rs61875074 | C | A | | 0.073 | | | 33.601 |  |
|  | -0.045 | 0.008 | 3.40E-08 | rs147895162 | C | T | | 0.015 | | | 30.481 |  |
|  | 0.014 | 0.003 | 3.30E-08 | rs10498638 | C | T | | 0.188 | | | 30.513 |  |
|  | -0.031 | 0.005 | 2.10E-08 | rs74805019 | C | G | | 0.034 | | | 31.386 |  |
|  | 0.037 | 0.006 | 2.70E-10 | rs74247887 | T | C | | 0.029 | | | 39.870 |  |
|  | -0.022 | 0.004 | 4.10E-08 | rs4788565 | A | G | | 0.067 | | | 30.111 |  |
|  | -0.024 | 0.004 | 4.00E-08 | rs7200852 | A | C | | 0.055 | | | 30.129 |  |
|  | 0.028 | 0.005 | 2.80E-08 | rs60304336 | T | G | | 0.041 | | | 30.842 |  |
| Nitrogen oxides | -0.015 | 0.003 | 3.60E-08 | rs7514956 | C | A | | 0.187 | | | 30.342 |  |
|  | -0.012 | 0.002 | 2.60E-08 | rs6749467 | A | G | | 0.465 | | | 30.958 |  |
|  | -0.014 | 0.003 | 4.80E-08 | rs1318845 | C | T | | 0.201 | | | 29.802 |  |
|  | -0.017 | 0.003 | 4.60E-09 | rs72808024 | C | A | | 0.148 | | | 34.336 |  |
|  | 0.019 | 0.002 | 3.20E-15 | rs12203592 | T | C | | 0.219 | | | 62.164 |  |
|  | 0.030 | 0.005 | 4.60E-08 | rs77255816 | T | C | | 0.037 | | | 29.880 |  |
|  | 0.013 | 0.002 | 1.00E-08 | rs77205736 | T | C | | 0.275 | | | 32.844 |  |
|  | 0.015 | 0.003 | 7.50E-09 | rs1217106 | G | A | | 0.782 | | | 33.405 |  |
| **East Asian** | | | | | | | | | | | |  |
| PM2.5 | -0.141 | 0.030 | 1.77E-06 | rs80151214 | G | A | | 0.616 | | | 22.834 |  |
|  | -0.134 | 0.029 | 2.69E-06 | rs7630570 | A | G | | 0.443 | | | 22.037 |  |
|  | -0.375 | 0.082 | 4.59E-06 | rs374130827 | A | G | | 0.969 | | | 21.004 |  |
|  | 0.457 | 0.099 | 4.12E-06 | rs11067476 | C | T | | 0.979 | | | 21.212 |  |
|  | 0.271 | 0.059 | 4.01E-06 | rs1608444 | T | A | | 0.932 | | | 21.258 |  |
| PM2.5-10 | -0.746 | 0.159 | 2.69E-06 | rs72888334 | G | T | | 0.987 | | | 22.013 |  |
|  | 0.700 | 0.153 | 4.61E-06 | rs2121854 | G | A | | 0.014 | | | 20.999 |  |
|  | 0.154 | 0.028 | 5.39E-08 | rs2319367 | T | C | | 0.527 | | | 29.560 |  |
|  | -0.158 | 0.034 | 2.56E-06 | rs12594133 | G | A | | 0.771 | | | 22.132 |  |
| PM10 | -0.450 | 0.091 | 7.24E-07 | rs62164084 | C | T | | 0.964 | | | 24.550 |  |
|  | -0.175 | 0.036 | 1.45E-06 | rs62365577 | C | G | | 0.802 | | | 23.220 |  |
|  | -0.153 | 0.032 | 1.45E-06 | rs201209537 | T | A | | 0.407 | | | 23.194 |  |
|  | -0.131 | 0.029 | 4.58E-06 | rs10097518 | G | A | | 0.377 | | | 21.012 |  |
|  | -0.164 | 0.034 | 1.66E-06 | rs17821828 | T | G | | 0.772 | | | 22.959 |  |
|  | -0.231 | 0.047 | 1.14E-06 | rs58880143 | G | A | | 0.867 | | | 23.688 |  |
|  | -0.135 | 0.028 | 1.97E-06 | rs6503238 | C | T | | 0.400 | | | 22.620 |  |
|  | -0.178 | 0.038 | 3.40E-06 | rs2424731 | C | T | | 0.173 | | | 21.568 |  |
| Nitrogen oxides | -0.144 | 0.030 | 1.49E-06 | rs4333856 | A | G | | 0.681 | | | 23.150 |  |
|  | -0.146 | 0.030 | 1.39E-06 | rs183491 | T | C | | 0.334 | | | 23.278 |  |
|  | 0.157 | 0.032 | 6.66E-07 | rs201266522 | A | G | | 0.498 | | | 24.700 |  |
|  | -0.160 | 0.032 | 8.15E-07 | rs11821352 | G | T | | 0.757 | | | 24.324 |  |
|  | 0.266 | 0.057 | 3.54E-06 | rs1608444 | T | A | | 0.931 | | | 21.493 |  |

PM: Particulate matter; SLE: Systemic lupus erythematosus; RA: Rheumatoid arthritis;CD: coeliac disease; MG: Myasthenia gravis; MSD: Multiple sclerosis disease;IBD: Inflammatory bowel disease

**Supplementary Table3.** The results of pleiotropy test, Cochrane’s Q between air pollution on ADs.

| **Exposure&Outcomes** | **Egger_intercept** | ***P* for pleiotropy** | **Cochrane’s Q** | ***P* for Cochrane’s Q** | **T-State** | | | ***P* for MR-PRESSO** | |
| --- | --- | --- | --- | --- | --- | --- | --- | --- | --- |
| **European** | | | | | | | | | |
| **PM2.5 on Ads** | |  |  |  |  | | | |  |
| SLE | -0.101 | 0.060 | 7.094 | 0.312 | 41.033 | 0.297 | | | |
| RA | -0.018 | 0.343 | 13.624 | 0.058 | 35.971 | 0.304 | | | |
| CD | 0.002 | 0.938 | 6.918 | 0.437 | 7.542 | 0.667 | | | |
| MG | 0.076 | 0.380 | 10.789 | 0.148 | 14.089 | 0.354 | | | |
| Psoriasis | 0.098 | 0.655 | **280.989** | **0.000** | **NA** | **<0.001** | | | |
| MSD | -0.026 | 0.417 | 4.663 | 0.701 | 6.094 | 0.790 | | | |
| IBD | 0.000 | 0.979 | 10.368 | 0.169 | 11.109 | 0.408 | | | |
| Vitiligo | -0.064 | 0.459 | 7.000 | 0.586 | 14.565 | 0.492 | | | |
| **PM2.5-10 on Ads** | |  |  |  |  |  | | | |
| SLE | 0.034 | 0.214 | 25.271 | 0.457 | 22.000 | 0.337 | | | |
| RA | 0.002 | 0.868 | **34.533** | **0.043** | 38.318 | 0.096 | | | |
| CD | 0.017 | 0.410 | 26.632 | 0.226 | 29.542 | 0.293 | | | |
| MG | -0.048 | 0.361 | 23.120 | 0.395 | 28.486 | 0.398 | | | |
| Psoriasis | -0.004 | 0.759 | 11.747 | 0.962 | 12.221 | 0.980 | | | |
| MSD | -0.010 | 0.718 | 26.169 | 0.244 | 29.776 | 0.286 | | | |
| IBD | 0.003 | 0.806 | 21.673 | 0.480 | 22.473 | 0.576 | | | |
| Vitiligo | -0.014 | 0.831 | 15.680 | 0.831 | 16.678 | 0.868 | | | |
| **PM10 on Ads** | |  |  |  |  |  | | | |
| SLE | -0.109 | 0.119 | **36.980** | **0.017** | **NA** | **<0.001** | | | |
| RA | 0.015 | 0.563 | **46.116** | **0.001** | **NA** | **<0.001** | | | |
| CD | -0.119 | 0.184 | **194.701** | **0.000** | **NA** | **<0.001** | | | |
| MG | -0.038 | 0.633 | 19.782 | 0.535 | 21.716 | 0.527 | | | |
| Psoriasis | -0.002 | 0.925 | 30.104 | 0.090 | 32.643 | 0.086 | | | |
| MSD | -0.004 | 0.927 | 20.022 | 0.520 | 21.810 | 0.533 | | | |
| IBD | -0.011 | 0.583 | 28.019 | 0.140 | 30.328 | 0.140 | | | |
| Vitiligo | -0.076 | 0.534 | 26.670 | 0.182 | 29.279 | 0.167 | | | |
| **NOx on Ads** | |  |  |  |  |  | | | |
| SLE | -0.049 | 0.885 | 8.037 | 0.090 | 11.949 | 0.139 | | | |
| RA | -0.026 | 0.720 | 8.324 | 0.305 | 11.685 | 0.295 | | | |
| CD | 0.045 | 0.784 | **14.246** | **0.047** | 19.489 | 0.064 | | | |
| MG | -0.544 | 0.358 | **26.744** | **0.000** | **NA** | **<0.001** | | | |
| Psoriasis | 0.073 | 0.324 | 4.783 | 0.686 | 6.600 | 0.680 | | | |
| MSD | -0.210 | 0.170 | 6.567 | 0.475 | 8.310 | 0.557 | | | |
| IBD | 0.040 | 0.683 | **14.525** | **0.043** | 19.150 | 0.067 | | | |
| Vitiligo | 0.432 | 0.293 | 4.544 | 0.715 | 6.347 | 0.680 | | | |
| **East Asian** | | | | | | | | | |
| **PM2.5 on Ads** | |  |  |  |  |  | | | |
| SLE | 0.053 | 0.358 | 2.279 | 0.517 | 4.224 | 0.556 | | | |
| RA | 0.007 | 0.868 | 2.101 | 0.552 | 3.259 | 0.650 | | | |
| **PM2.5-10 on Ads** | |  |  |  |  |  | | | |
| SLE | 0.053 | 0.358 | 2.279 | 0.517 | 4.224 | 0.499 | | | |
| RA | NA | NA | 2.932 | 0.087 | 3.259 | 0.650 | | | |
| **PM10 on Ads** | |  |  |  |  |  | | | |
| SLE | -0.021 | 0.522 | 1.910 | 0.752 | 3.014 | 0.778 | | | |
| RA | 0.039 | 0.390 | **11.132** | **0.025** | 25.086 | 0.220 | | | |
| **NOx on Ads** | |  |  |  |  |  | | | |
| SLE | -0.722 | 0.432 | 5.019 | 0.081 | **NA** | | **NA** | | |
| RA | 0.117 | 0.729 | 1.752 | 0.625 | **3.041** | | **0.644** | | |

Ads: Autoimmune diseases; PM: Particulate matter; SLE: Systemic lupus erythematosus; RA: Rheumatoid arthritis;CD: coeliac disease; MG: Myasthenia gravis; MSD: Multiple sclerosis disease;IBD: Inflammatory bowel disease; NOx: Nitrogen Oxides.

**Supplementary Table 4.** Excluded SNPs of P<0.05 for MR-PRESSO (European population)

| **Filter through MR-PRESSO** | **Exclude SNPs** |
| --- | --- |
| **PM2.5 on Psoriasis** | rs114708313 |
|  | rs72642437 |
| **PM10 on SLE** | rs2248162 |
| **PM10 on RA** | rs2248162 |
| **PM10 on CD** | rs2248162 |

PM: Particulate matter; SLE: Systemic lupus erythematosus; RA: Rheumatoid arthritis;CD: coeliac disease;

**Supplementary Table 5.** The leave-one-out analysis for air pollution on ADs (European population)

| **PM2.5 on Ads** | **SNP** | **BETA** | **SE** | ***P*-value** |
| --- | --- | --- | --- | --- |
| **Systemic lupus erythematosus** | rs114708313 | -2.626 | 4.695 | 0.576 |
|  | rs12203592 | -12.208 | 8.179 | 0.136 |
|  | rs1372504 | 1.554 | 5.231 | 0.766 |
|  | rs1537371 | -6.119 | 4.996 | 0.221 |
|  | rs6749467 | -7.311 | 5.027 | 0.146 |
|  | rs72642437 | 3.290 | 1.140 | 0.004 |
|  | rs77205736 | 1.908 | 6.959 | 0.784 |
|  | rs77255816 | 16.092 | 9.792 | 0.100 |
|  | ALL | 2.005 | 1.401 | 0.152 |
| **Rheumatoid arthritis** | rs114708313 | -4.247 | 1.486 | 0.004 |
|  | rs12203592 | -1.699 | 2.732 | 0.534 |
|  | rs1372504 | 1.172 | 1.676 | 0.485 |
|  | rs1537371 | -2.352 | 1.592 | 0.140 |
|  | rs6749467 | 0.500 | 1.606 | 0.755 |
|  | rs72642437 | 0.549 | 0.354 | 0.120 |
|  | rs77205736 | 0.067 | 2.152 | 0.975 |
|  | rs77255816 | 1.867 | 3.204 | 0.560 |
|  | ALL | 0.212 | 0.440 | 0.631 |
| **Coeliac disease** | rs114708313 | -1.987 | 2.712 | 0.464 |
|  | rs12203592 | -5.857 | 4.694 | 0.212 |
|  | rs1372504 | 2.115 | 2.913 | 0.468 |
|  | rs1537371 | -3.120 | 2.781 | 0.262 |
|  | rs6749467 | 3.986 | 2.800 | 0.155 |
|  | rs72642437 | -0.196 | 0.616 | 0.750 |
|  | rs77205736 | -3.136 | 3.661 | 0.392 |
|  | rs77255816 | 3.536 | 5.721 | 0.537 |
|  | ALL | -0.250 | 0.550 | 0.650 |
| **Myasthenia gravis** | rs114708313 | 7.676 | 7.020 | 0.274 |
|  | rs12203592 | -18.734 | 12.610 | 0.137 |
|  | rs1372504 | 7.501 | 7.949 | 0.345 |
|  | rs1537371 | -9.903 | 7.566 | 0.191 |
|  | rs6749467 | -2.284 | 7.634 | 0.765 |
|  | rs72642437 | 2.192 | 1.713 | 0.201 |
|  | rs77205736 | -13.038 | 10.568 | 0.217 |
|  | rs77255816 | 24.269 | 15.013 | 0.106 |
|  | ALL | 1.583 | 1.890 | 0.402 |
| **Psoriasis** | rs12203592 | -1.888 | 3.060 | 0.537 |
|  | rs1372504 | 1.367 | 1.920 | 0.477 |
|  | rs1537371 | 1.164 | 1.827 | 0.524 |
|  | rs6749467 | 0.113 | 1.840 | 0.951 |
|  | rs77205736 | -0.762 | 2.418 | 0.753 |
|  | rs77255816 | -4.504 | 3.695 | 0.223 |
|  | ALL | 0.075 | 0.906 | 0.934 |
| **Multiple sclerosis disease** | rs114708313 | -1.702 | 3.473 | 0.624 |
|  | rs12203592 | -3.060 | 6.023 | 0.611 |
|  | rs1372504 | -2.058 | 3.848 | 0.593 |
|  | rs1537371 | -4.284 | 3.678 | 0.244 |
|  | rs6749467 | -3.002 | 3.696 | 0.417 |
|  | rs72642437 | -0.926 | 0.832 | 0.266 |
|  | rs77205736 | -1.390 | 4.888 | 0.776 |
|  | rs77255816 | 12.324 | 7.275 | 0.090 |
|  | ALL | -1.127 | 0.739 | 0.127 |
| **Inflammatory bowel disease** | rs114708313 | 3.498 | 1.494 | 0.019 |
|  | rs12203592 | -6.056 | 2.746 | 0.027 |
|  | rs1372504 | 0.911 | 1.709 | 0.594 |
|  | rs1537371 | 0.259 | 1.625 | 0.874 |
|  | rs6749467 | 0.621 | 1.638 | 0.704 |
|  | rs72642437 | 0.449 | 0.360 | 0.212 |
|  | rs77205736 | -1.043 | 2.182 | 0.633 |
|  | rs77255816 | -0.194 | 3.290 | 0.953 |
|  | ALL | 0.478 | 0.391 | 0.221 |
| **Vitiligo** | rs114708313 | -15.331 | 9.268 | 0.098 |
|  | rs12203592 | -6.822 | 17.124 | 0.690 |
|  | rs1372504 | -6.224 | 10.617 | 0.558 |
|  | rs1537371 | -0.873 | 10.137 | 0.931 |
|  | rs6749467 | 6.585 | 10.216 | 0.519 |
|  | rs72642437 | 3.566 | 2.243 | 0.112 |
|  | rs77205736 | 10.768 | 13.992 | 0.442 |
|  | rs77255816 | -4.367 | 20.354 | 0.830 |
|  | ALL | 2.208 | 2.002 | 0.270 |
| **PM2.5-10 on Ads** | **SNP** | **BETA** | **SE** | ***P*-value** |
| **Systemic lupus erythematosus** | rs10152521 | -0.377 | 1.145 | 0.742 |
|  | rs111308789 | -0.251 | 6.236 | 0.968 |
|  | rs1157546 | -5.337 | 6.393 | 0.404 |
|  | rs11621531 | -5.768 | 6.775 | 0.395 |
|  | rs116259145 | -4.413 | 5.851 | 0.451 |
|  | rs116816317 | 1.952 | 8.114 | 0.810 |
|  | rs117125329 | 5.911 | 5.244 | 0.260 |
|  | rs117389221 | 3.128 | 6.034 | 0.604 |
|  | rs118101191 | 5.673 | 8.767 | 0.518 |
|  | rs12462492 | -6.166 | 5.318 | 0.246 |
|  | rs13125748 | -3.039 | 6.173 | 0.623 |
|  | rs138141967 | -7.348 | 8.820 | 0.405 |
|  | rs1706918 | -6.137 | 6.213 | 0.323 |
|  | rs17675316 | 3.631 | 5.108 | 0.477 |
|  | rs57048268 | -0.917 | 6.770 | 0.892 |
|  | rs605027 | -11.664 | 5.977 | 0.051 |
|  | rs62079137 | 13.175 | 6.444 | 0.041 |
|  | rs71323440 | 7.046 | 6.991 | 0.314 |
|  | rs76170056 | -13.672 | 6.918 | 0.048 |
|  | rs78060907 | 9.374 | 8.396 | 0.264 |
|  | rs8006373 | 5.289 | 5.181 | 0.307 |
|  | rs8051340 | -7.366 | 6.004 | 0.220 |
|  | rs9497937 | 0.070 | 5.387 | 0.990 |
|  | ALL | -0.551 | 0.912 | 0.546 |
| **Rheumatoid arthritis** | rs10152521 | -0.014 | 0.364 | 0.969 |
|  | rs111308789 | -0.893 | 2.010 | 0.657 |
|  | rs1157546 | 0.780 | 2.036 | 0.702 |
|  | rs11621531 | 0.813 | 2.179 | 0.709 |
|  | rs116259145 | 2.997 | 1.827 | 0.101 |
|  | rs116816317 | 1.610 | 2.624 | 0.540 |
|  | rs117125329 | -1.441 | 1.771 | 0.416 |
|  | rs117389221 | 0.353 | 1.895 | 0.852 |
|  | rs118101191 | 9.796 | 2.828 | 0.001 |
|  | rs12462492 | 0.807 | 1.703 | 0.636 |
|  | rs13125748 | 3.161 | 1.973 | 0.109 |
|  | rs138141967 | 0.189 | 2.934 | 0.949 |
|  | rs1706918 | 2.229 | 1.984 | 0.261 |
|  | rs17675316 | 3.956 | 1.618 | 0.014 |
|  | rs57048268 | -2.123 | 2.178 | 0.330 |
|  | rs605027 | 1.185 | 1.896 | 0.532 |
|  | rs62079137 | -0.968 | 2.086 | 0.643 |
|  | rs71323440 | -3.560 | 2.259 | 0.115 |
|  | rs76170056 | -2.126 | 2.211 | 0.336 |
|  | rs78060907 | -2.709 | 2.623 | 0.302 |
|  | rs8006373 | 1.905 | 1.644 | 0.246 |
|  | rs8051340 | -2.501 | 1.910 | 0.190 |
|  | rs9497937 | -0.133 | 1.715 | 0.938 |
|  | ALL | 0.255 | 0.347 | 0.462 |
| **Coeliac disease** | rs10152521 | -1.062 | 0.632 | 0.093 |
|  | rs111308789 | -2.799 | 3.469 | 0.420 |
|  | rs1157546 | -5.329 | 3.528 | 0.131 |
|  | rs11621531 | 5.240 | 3.766 | 0.164 |
|  | rs116259145 | 0.050 | 3.232 | 0.988 |
|  | rs116816317 | 0.845 | 4.539 | 0.852 |
|  | rs117125329 | 2.880 | 3.198 | 0.368 |
|  | rs117389221 | -4.018 | 3.289 | 0.222 |
|  | rs118101191 | 0.813 | 5.128 | 0.874 |
|  | rs12462492 | -0.105 | 2.954 | 0.972 |
|  | rs13125748 | -2.925 | 3.449 | 0.396 |
|  | rs138141967 | -2.769 | 4.944 | 0.575 |
|  | rs1706918 | 9.663 | 3.450 | 0.005 |
|  | rs17675316 | -4.409 | 2.870 | 0.124 |
|  | rs57048268 | -3.013 | 3.793 | 0.427 |
|  | rs605027 | 1.852 | 3.309 | 0.576 |
|  | rs62079137 | 2.438 | 3.625 | 0.501 |
|  | rs71323440 | -1.645 | 3.928 | 0.675 |
|  | rs76170056 | 3.180 | 3.847 | 0.408 |
|  | rs78060907 | -2.218 | 4.512 | 0.623 |
|  | rs8006373 | 4.749 | 2.883 | 0.100 |
|  | rs8051340 | -4.598 | 3.341 | 0.169 |
|  | rs9497937 | -1.308 | 2.991 | 0.662 |
|  | ALL | -0.675 | 0.531 | 0.203 |
| **Myasthenia gravis** | rs10152521 | 3.285 | 1.735 | 0.058 |
|  | rs111308789 | 2.329 | 9.508 | 0.806 |
|  | rs1157546 | 13.937 | 9.799 | 0.155 |
|  | rs11621531 | -10.100 | 10.282 | 0.326 |
|  | rs116259145 | 12.333 | 8.799 | 0.161 |
|  | rs116816317 | 13.820 | 12.571 | 0.272 |
|  | rs117125329 | -5.550 | 8.206 | 0.499 |
|  | rs117389221 | -6.710 | 8.948 | 0.453 |
|  | rs118101191 | 4.114 | 13.432 | 0.759 |
|  | rs12462492 | 5.689 | 8.070 | 0.481 |
|  | rs13125748 | -5.300 | 9.369 | 0.572 |
|  | rs138141967 | -6.752 | 13.440 | 0.615 |
|  | rs1706918 | -2.927 | 9.445 | 0.757 |
|  | rs17675316 | -12.378 | 7.771 | 0.111 |
|  | rs57048268 | -3.122 | 10.318 | 0.762 |
|  | rs605027 | -4.257 | 9.023 | 0.637 |
|  | rs62079137 | -9.516 | 9.838 | 0.333 |
|  | rs71323440 | 13.214 | 10.630 | 0.214 |
|  | rs76170056 | 21.408 | 10.510 | 0.042 |
|  | rs78060907 | -11.105 | 12.669 | 0.381 |
|  | rs8006373 | -7.837 | 7.820 | 0.316 |
|  | rs8051340 | -8.922 | 9.077 | 0.326 |
|  | rs9497937 | 4.087 | 8.143 | 0.616 |
|  | ALL | 1.580 | 1.352 | 0.243 |
| **Psoriasis** | rs10152521 | -0.142 | 0.419 | 0.734 |
|  | rs111308789 | 0.907 | 2.302 | 0.694 |
|  | rs1157546 | 0.492 | 2.309 | 0.831 |
|  | rs11621531 | -1.297 | 2.482 | 0.601 |
|  | rs116259145 | -2.165 | 2.105 | 0.304 |
|  | rs116816317 | 2.574 | 2.985 | 0.389 |
|  | rs117125329 | 0.725 | 2.059 | 0.725 |
|  | rs117389221 | 0.303 | 2.168 | 0.889 |
|  | rs118101191 | 3.516 | 3.300 | 0.287 |
|  | rs12462492 | -1.340 | 1.945 | 0.491 |
|  | rs13125748 | -0.227 | 2.253 | 0.920 |
|  | rs138141967 | 3.330 | 3.300 | 0.313 |
|  | rs1706918 | -2.698 | 2.284 | 0.237 |
|  | rs17675316 | -2.550 | 1.889 | 0.177 |
|  | rs57048268 | -0.082 | 2.486 | 0.974 |
|  | rs605027 | 0.290 | 2.177 | 0.894 |
|  | rs62079137 | 1.130 | 2.380 | 0.635 |
|  | rs71323440 | -1.626 | 2.566 | 0.526 |
|  | rs76170056 | -1.684 | 2.538 | 0.507 |
|  | rs78060907 | -1.374 | 2.998 | 0.647 |
|  | rs8006373 | 0.102 | 1.894 | 0.957 |
|  | rs8051340 | 3.185 | 2.190 | 0.146 |
|  | rs9497937 | -0.947 | 1.965 | 0.630 |
|  | ALL | -0.172 | 0.318 | 0.590 |
| **Multiple sclerosis disease** | rs10152521 | 0.268 | 0.837 | 0.749 |
|  | rs111308789 | -3.446 | 4.576 | 0.451 |
|  | rs1157546 | 8.942 | 4.703 | 0.057 |
|  | rs11621531 | -0.156 | 4.998 | 0.975 |
|  | rs116259145 | 4.393 | 4.267 | 0.303 |
|  | rs116816317 | 16.526 | 5.936 | 0.005 |
|  | rs117125329 | 2.210 | 3.953 | 0.576 |
|  | rs117389221 | 4.398 | 4.458 | 0.324 |
|  | rs118101191 | 9.886 | 6.551 | 0.131 |
|  | rs12462492 | 1.380 | 3.898 | 0.723 |
|  | rs13125748 | -3.763 | 4.532 | 0.406 |
|  | rs138141967 | 7.737 | 6.526 | 0.236 |
|  | rs1706918 | -1.264 | 4.572 | 0.782 |
|  | rs17675316 | 7.338 | 3.761 | 0.051 |
|  | rs57048268 | -0.708 | 4.973 | 0.887 |
|  | rs605027 | 2.431 | 4.380 | 0.579 |
|  | rs62079137 | -3.856 | 4.743 | 0.416 |
|  | rs71323440 | 4.542 | 5.107 | 0.374 |
|  | rs76170056 | -6.009 | 5.082 | 0.237 |
|  | rs78060907 | -0.357 | 6.154 | 0.954 |
|  | rs8006373 | -0.455 | 3.805 | 0.905 |
|  | rs8051340 | -5.419 | 4.417 | 0.220 |
|  | rs9497937 | 3.383 | 3.954 | 0.392 |
|  | ALL | 0.907 | 0.695 | 0.192 |
| **Inflammatory bowel disease** | rs10152521 | -0.098 | 0.370 | 0.791 |
|  | rs111308789 | 0.980 | 2.038 | 0.631 |
|  | rs1157546 | -0.658 | 2.077 | 0.751 |
|  | rs11621531 | -3.096 | 2.201 | 0.160 |
|  | rs116259145 | -2.085 | 1.866 | 0.264 |
|  | rs116816317 | -5.602 | 2.699 | 0.038 |
|  | rs117125329 | -1.590 | 1.847 | 0.389 |
|  | rs117389221 | -0.368 | 1.919 | 0.848 |
|  | rs118101191 | 1.786 | 2.944 | 0.544 |
|  | rs12462492 | 1.041 | 1.727 | 0.547 |
|  | rs13125748 | 1.886 | 2.008 | 0.348 |
|  | rs138141967 | 3.906 | 2.937 | 0.184 |
|  | rs1706918 | -1.035 | 2.027 | 0.610 |
|  | rs17675316 | 1.423 | 1.680 | 0.397 |
|  | rs57048268 | -1.996 | 2.214 | 0.367 |
|  | rs605027 | 2.712 | 1.931 | 0.160 |
|  | rs62079137 | 0.317 | 2.115 | 0.881 |
|  | rs71323440 | -0.669 | 2.289 | 0.770 |
|  | rs76170056 | -4.459 | 2.247 | 0.047 |
|  | rs78060907 | 2.378 | 2.657 | 0.371 |
|  | rs8006373 | 1.496 | 1.672 | 0.371 |
|  | rs8051340 | -0.915 | 1.941 | 0.638 |
|  | rs9497937 | -0.736 | 1.746 | 0.673 |
|  | ALL | -0.136 | 0.282 | 0.630 |
| **Vitiligo** | rs10152521 | 4.930 | 2.301 | 0.032 |
|  | rs111308789 | 1.942 | 12.758 | 0.879 |
|  | rs1157546 | 5.463 | 12.933 | 0.673 |
|  | rs11621531 | 6.131 | 13.832 | 0.658 |
|  | rs116259145 | 0.690 | 11.799 | 0.953 |
|  | rs116816317 | 32.057 | 16.909 | 0.058 |
|  | rs117125329 | -4.693 | 11.355 | 0.679 |
|  | rs117389221 | -14.485 | 11.944 | 0.225 |
|  | rs118101191 | 22.679 | 18.332 | 0.216 |
|  | rs12462492 | 2.776 | 10.798 | 0.797 |
|  | rs13125748 | -4.497 | 12.521 | 0.719 |
|  | rs138141967 | -16.305 | 18.184 | 0.370 |
|  | rs1706918 | 21.244 | 12.687 | 0.094 |
|  | rs17675316 | -0.555 | 10.362 | 0.957 |
|  | rs57048268 | 14.256 | 13.839 | 0.303 |
|  | rs605027 | -3.107 | 12.059 | 0.797 |
|  | rs62079137 | 9.014 | 13.187 | 0.494 |
|  | rs71323440 | -7.156 | 14.264 | 0.616 |
|  | rs76170056 | -9.365 | 13.963 | 0.502 |
|  | rs78060907 | 13.794 | 16.789 | 0.411 |
|  | rs8006373 | 11.897 | 10.464 | 0.256 |
|  | rs8051340 | -6.582 | 12.138 | 0.588 |
|  | rs9497937 | 12.559 | 10.884 | 0.249 |
|  | ALL | 4.222 | 1.757 | 0.016 |
| **PM10 on Ads** | **SNP** | **BETA** | **SE** | ***P*-value** |
| **Systemic lupus erythematosus** | rs10498638 | -6.936 | 5.737 | 0.227 |
|  | rs114789974 | -4.963 | 4.336 | 0.252 |
|  | rs13084230 | -3.761 | 5.051 | 0.457 |
|  | rs13122455 | 1.700 | 5.320 | 0.749 |
|  | rs140295641 | 0.968 | 10.374 | 0.926 |
|  | rs142169179 | 19.082 | 6.928 | 0.006 |
|  | rs147895162 | 1.117 | 5.072 | 0.826 |
|  | rs182549 | -11.140 | 5.039 | 0.027 |
|  | rs2004679 | 2.107 | 7.428 | 0.777 |
|  | rs4788565 | 3.791 | 4.434 | 0.393 |
|  | rs4833095 | -5.699 | 3.564 | 0.110 |
|  | rs56084453 | 2.658 | 4.908 | 0.588 |
|  | rs60304336 | -3.204 | 7.930 | 0.686 |
|  | rs61620752 | -3.827 | 5.004 | 0.444 |
|  | rs61875074 | -0.031 | 4.536 | 0.994 |
|  | rs6793835 | 1.178 | 5.783 | 0.839 |
|  | rs6867849 | 1.290 | 5.134 | 0.802 |
|  | rs7200852 | 11.249 | 9.575 | 0.240 |
|  | rs74247887 | 7.314 | 6.943 | 0.292 |
|  | rs74805019 | -6.128 | 4.369 | 0.161 |
|  | rs9640029 | -1.608 | 4.449 | 0.718 |
|  | ALL | -1.189 | 1.254 | 0.343 |
| **Rheumatoid arthritis** | rs10498638 | 4.795 | 1.834 | 0.009 |
|  | rs114789974 | 0.558 | 1.388 | 0.688 |
|  | rs13084230 | -0.715 | 1.607 | 0.656 |
|  | rs13122455 | -0.764 | 1.700 | 0.653 |
|  | rs140295641 | 1.400 | 3.512 | 0.690 |
|  | rs142169179 | 5.305 | 2.227 | 0.017 |
|  | rs147895162 | -0.018 | 1.595 | 0.991 |
|  | rs182549 | -1.054 | 1.602 | 0.510 |
|  | rs2004679 | -1.595 | 2.291 | 0.486 |
|  | rs4788565 | -1.136 | 1.391 | 0.414 |
|  | rs4833095 | 0.171 | 1.175 | 0.884 |
|  | rs56084453 | -0.295 | 1.567 | 0.851 |
|  | rs60304336 | -0.748 | 2.567 | 0.771 |
|  | rs61620752 | 1.637 | 1.599 | 0.306 |
|  | rs61875074 | 0.624 | 1.446 | 0.666 |
|  | rs6793835 | 3.165 | 1.871 | 0.091 |
|  | rs6867849 | 3.040 | 1.595 | 0.057 |
|  | rs7200852 | -2.075 | 3.152 | 0.510 |
|  | rs74247887 | 1.231 | 2.262 | 0.586 |
|  | rs74805019 | -0.068 | 1.384 | 0.961 |
|  | rs9640029 | -0.203 | 1.420 | 0.886 |
|  | ALL | 0.519 | 0.379 | 0.171 |
| **Coeliac disease** | rs10498638 | 2.555 | 3.190 | 0.423 |
|  | rs114789974 | 1.224 | 2.424 | 0.613 |
|  | rs13084230 | -0.479 | 2.787 | 0.863 |
|  | rs13122455 | -1.935 | 2.949 | 0.512 |
|  | rs140295641 | 6.444 | 6.071 | 0.289 |
|  | rs142169179 | -1.956 | 3.902 | 0.616 |
|  | rs147895162 | -4.665 | 2.799 | 0.096 |
|  | rs182549 | -3.204 | 2.801 | 0.253 |
|  | rs2004679 | -3.609 | 3.911 | 0.356 |
|  | rs4788565 | -3.787 | 2.423 | 0.118 |
|  | rs4833095 | -0.370 | 2.023 | 0.855 |
|  | rs56084453 | 2.658 | 2.739 | 0.332 |
|  | rs60304336 | 3.083 | 4.586 | 0.502 |
|  | rs61620752 | -1.027 | 2.776 | 0.711 |
|  | rs61875074 | -2.591 | 2.524 | 0.305 |
|  | rs6793835 | 4.667 | 3.242 | 0.150 |
|  | rs6867849 | -0.426 | 2.828 | 0.880 |
|  | rs7200852 | 0.327 | 5.440 | 0.952 |
|  | rs74247887 | 5.095 | 4.007 | 0.204 |
|  | rs74805019 | 1.332 | 2.432 | 0.584 |
|  | rs9640029 | 0.826 | 2.471 | 0.738 |
|  | ALL | -0.290 | 0.638 | 0.649 |
| **Myasthenia gravis** | rs10498638 | 2.127 | 8.677 | 0.806 |
|  | rs114789974 | 6.429 | 6.644 | 0.333 |
|  | rs13084230 | 1.622 | 7.646 | 0.832 |
|  | rs13122455 | 11.268 | 8.048 | 0.161 |
|  | rs140295641 | 21.050 | 15.995 | 0.188 |
|  | rs142169179 | 11.051 | 10.350 | 0.286 |
|  | rs147895162 | 4.459 | 7.672 | 0.561 |
|  | rs182549 | 10.521 | 7.639 | 0.168 |
|  | rs2004679 | -9.593 | 11.238 | 0.393 |
|  | rs2248162 | -10.678 | 8.487 | 0.208 |
|  | rs4788565 | 2.582 | 6.720 | 0.701 |
|  | rs4833095 | -5.902 | 5.472 | 0.281 |
|  | rs56084453 | -7.580 | 7.439 | 0.308 |
|  | rs60304336 | -20.801 | 12.062 | 0.085 |
|  | rs61620752 | 0.286 | 7.580 | 0.970 |
|  | rs61875074 | -8.340 | 6.871 | 0.225 |
|  | rs6793835 | 3.596 | 8.848 | 0.684 |
|  | rs6867849 | 7.711 | 7.666 | 0.315 |
|  | rs7200852 | -0.589 | 14.610 | 0.968 |
|  | rs74247887 | 5.502 | 10.608 | 0.604 |
|  | rs74805019 | 3.324 | 6.567 | 0.613 |
|  | rs9640029 | 8.375 | 6.738 | 0.214 |
|  | ALL | 1.408 | 1.702 | 0.408 |
| **Psoriasis** | rs10498638 | 0.343 | 2.105 | 0.871 |
|  | rs114789974 | 0.540 | 1.601 | 0.736 |
|  | rs13084230 | 0.369 | 1.836 | 0.841 |
|  | rs13122455 | -0.407 | 1.949 | 0.835 |
|  | rs140295641 | 4.318 | 3.950 | 0.274 |
|  | rs142169179 | -1.665 | 2.528 | 0.510 |
|  | rs147895162 | -0.389 | 1.836 | 0.832 |
|  | rs182549 | -2.970 | 1.843 | 0.107 |
|  | rs2004679 | -2.543 | 2.585 | 0.325 |
|  | rs2248162 | 7.497 | 2.065 | 0.000 |
|  | rs4788565 | -0.411 | 1.606 | 0.798 |
|  | rs4833095 | 0.143 | 1.326 | 0.914 |
|  | rs56084453 | -1.058 | 1.788 | 0.554 |
|  | rs60304336 | -3.165 | 2.936 | 0.281 |
|  | rs61620752 | -1.662 | 1.836 | 0.365 |
|  | rs61875074 | 0.135 | 1.653 | 0.935 |
|  | rs6793835 | 0.200 | 2.125 | 0.925 |
|  | rs6867849 | -0.836 | 1.836 | 0.649 |
|  | rs7200852 | -6.423 | 3.553 | 0.071 |
|  | rs74247887 | 2.397 | 2.599 | 0.356 |
|  | rs74805019 | 3.468 | 1.595 | 0.030 |
|  | rs9640029 | 0.355 | 1.623 | 0.827 |
|  | ALL | 0.104 | 0.491 | 0.833 |
| **Multiple sclerosis disease** | rs10498638 | -2.712 | 4.217 | 0.520 |
|  | rs114789974 | 0.489 | 3.188 | 0.878 |
|  | rs13084230 | 1.777 | 3.709 | 0.632 |
|  | rs13122455 | 4.077 | 3.913 | 0.297 |
|  | rs140295641 | -10.369 | 7.693 | 0.178 |
|  | rs142169179 | 1.530 | 5.057 | 0.762 |
|  | rs147895162 | -2.361 | 3.733 | 0.527 |
|  | rs182549 | 5.272 | 3.703 | 0.154 |
|  | rs2004679 | 0.462 | 5.229 | 0.930 |
|  | rs2248162 | -11.033 | 4.087 | 0.007 |
|  | rs4788565 | 2.172 | 3.235 | 0.502 |
|  | rs4833095 | 1.414 | 2.620 | 0.590 |
|  | rs56084453 | -2.732 | 3.596 | 0.447 |
|  | rs60304336 | 4.937 | 5.828 | 0.397 |
|  | rs61620752 | 2.713 | 3.666 | 0.459 |
|  | rs61875074 | -1.976 | 3.323 | 0.552 |
|  | rs6793835 | -2.811 | 4.235 | 0.507 |
|  | rs6867849 | -5.798 | 3.758 | 0.123 |
|  | rs7200852 | -0.426 | 7.070 | 0.952 |
|  | rs74247887 | 4.015 | 5.085 | 0.430 |
|  | rs74805019 | 0.469 | 3.204 | 0.884 |
|  | rs9640029 | 1.594 | 3.268 | 0.626 |
|  | ALL | -0.073 | 0.822 | 0.929 |
| **Inflammatory bowel disease** | rs10498638 | -0.250 | 1.862 | 0.893 |
|  | rs114789974 | 2.271 | 1.418 | 0.109 |
|  | rs13084230 | -0.612 | 1.630 | 0.707 |
|  | rs13122455 | -0.921 | 1.728 | 0.594 |
|  | rs140295641 | -4.773 | 3.509 | 0.174 |
|  | rs142169179 | -4.084 | 2.255 | 0.070 |
|  | rs147895162 | 2.853 | 1.631 | 0.080 |
|  | rs182549 | -2.383 | 1.634 | 0.145 |
|  | rs2004679 | -0.050 | 2.325 | 0.983 |
|  | rs2248162 | 4.349 | 1.819 | 0.017 |
|  | rs4788565 | -1.483 | 1.423 | 0.298 |
|  | rs4833095 | 1.016 | 1.183 | 0.391 |
|  | rs56084453 | 0.134 | 1.594 | 0.933 |
|  | rs60304336 | -3.032 | 2.624 | 0.248 |
|  | rs61620752 | -1.662 | 1.624 | 0.306 |
|  | rs61875074 | 0.885 | 1.464 | 0.546 |
|  | rs6793835 | 0.970 | 1.894 | 0.609 |
|  | rs6867849 | -0.457 | 1.633 | 0.779 |
|  | rs7200852 | 6.431 | 3.197 | 0.044 |
|  | rs74247887 | 0.811 | 2.313 | 0.726 |
|  | rs74805019 | 0.378 | 1.416 | 0.790 |
|  | rs9640029 | 0.514 | 1.442 | 0.721 |
|  | ALL | 0.202 | 0.421 | 0.632 |
| **Vitiligo** | rs10498638 | 3.083 | 11.632 | 0.791 |
|  | rs114789974 | 18.822 | 8.792 | 0.032 |
|  | rs13084230 | -10.301 | 10.175 | 0.311 |
|  | rs13122455 | 15.539 | 10.797 | 0.150 |
|  | rs140295641 | 13.388 | 21.841 | 0.540 |
|  | rs142169179 | 1.839 | 14.045 | 0.896 |
|  | rs147895162 | 10.521 | 10.136 | 0.299 |
|  | rs182549 | -3.590 | 10.191 | 0.725 |
|  | rs2004679 | 7.436 | 14.847 | 0.616 |
|  | rs2248162 | 24.698 | 11.313 | 0.029 |
|  | rs4788565 | -10.347 | 8.887 | 0.244 |
|  | rs4833095 | 10.474 | 7.415 | 0.158 |
|  | rs56084453 | -4.131 | 9.936 | 0.678 |
|  | rs60304336 | 29.619 | 16.318 | 0.070 |
|  | rs61620752 | -9.042 | 10.150 | 0.373 |
|  | rs61875074 | -4.994 | 9.175 | 0.586 |
|  | rs6793835 | -9.934 | 11.867 | 0.403 |
|  | rs6867849 | 8.425 | 10.144 | 0.406 |
|  | rs7200852 | -26.134 | 19.883 | 0.189 |
|  | rs74247887 | -20.050 | 14.543 | 0.168 |
|  | rs74805019 | -2.608 | 8.752 | 0.766 |
|  | rs9640029 | 7.622 | 9.013 | 0.398 |
|  | ALL | 2.712 | 2.564 | 0.290 |
| **NOx on Ads** | **SNP** | **BETA** | **SE** | ***P*-value** |
| **Systemic lupus erythematosus** | rs10983735 | -4.770 | 6.217 | 0.443 |
|  | rs12203592 | -16.592 | 11.116 | 0.136 |
|  | rs34623735 | 10.181 | 5.448 | 0.062 |
|  | rs7225402 | 9.670 | 5.190 | 0.062 |
|  | rs77205736 | 1.675 | 6.108 | 0.784 |
|  | ALL | 3.717 | 3.908 | 0.341 |
| **Rheumatoid arthritis** | rs1217106 | -0.618 | 1.724 | 0.720 |
|  | rs12203592 | -1.900 | 3.056 | 0.534 |
|  | rs1318845 | -4.586 | 1.651 | 0.005 |
|  | rs6749467 | 0.532 | 1.707 | 0.755 |
|  | rs72808024 | 1.251 | 1.674 | 0.455 |
|  | rs7514956 | -0.739 | 1.636 | 0.651 |
|  | rs77205736 | 0.068 | 2.194 | 0.975 |
|  | rs77255816 | 1.960 | 3.364 | 0.560 |
|  | ALL | -0.712 | 0.738 | 0.335 |
| **Coeliac disease** | rs1217106 | -7.962 | 2.974 | 0.007 |
|  | rs12203592 | -6.551 | 5.250 | 0.212 |
|  | rs1318845 | -1.686 | 2.872 | 0.557 |
|  | rs6749467 | 4.236 | 2.976 | 0.155 |
|  | rs72808024 | 1.967 | 2.942 | 0.504 |
|  | rs7514956 | 3.751 | 2.861 | 0.190 |
|  | rs77205736 | -3.196 | 3.732 | 0.392 |
|  | rs77255816 | 3.712 | 6.006 | 0.537 |
|  | ALL | -0.423 | 1.680 | 0.801 |
| **Myasthenia gravis** | rs1217106 | -12.523 | 8.147 | 0.124 |
|  | rs12203592 | -20.955 | 14.104 | 0.137 |
|  | rs6749467 | -2.427 | 8.112 | 0.765 |
|  | rs7514956 | -2.108 | 7.768 | 0.786 |
|  | rs77205736 | -13.291 | 10.773 | 0.217 |
|  | rs77255816 | 25.477 | 15.760 | 0.106 |
|  | ALL | -5.857 | 4.543 | 0.197 |
| **Psoriasis** | rs1217106 | -1.470 | 1.965 | 0.454 |
|  | rs12203592 | -2.112 | 3.423 | 0.537 |
|  | rs1318845 | -4.064 | 1.891 | 0.032 |
|  | rs6749467 | 0.120 | 1.955 | 0.951 |
|  | rs72808024 | -1.826 | 1.920 | 0.342 |
|  | rs7514956 | 0.739 | 1.875 | 0.693 |
|  | rs77205736 | -0.776 | 2.465 | 0.753 |
|  | rs77255816 | -4.728 | 3.879 | 0.223 |
|  | ALL | -1.431 | 0.773 | 0.064 |
| **Multiple sclerosis disease** | rs1217106 | 6.553 | 3.943 | 0.097 |
|  | rs12203592 | -3.423 | 6.737 | 0.611 |
|  | rs1318845 | 3.655 | 3.796 | 0.336 |
|  | rs6749467 | -3.190 | 3.928 | 0.417 |
|  | rs72808024 | 2.860 | 3.870 | 0.460 |
|  | rs7514956 | 0.938 | 3.778 | 0.804 |
|  | rs77205736 | -1.417 | 4.983 | 0.776 |
|  | rs77255816 | 12.937 | 7.637 | 0.090 |
|  | ALL | 1.963 | 1.553 | 0.206 |
| **Inflammatory bowel disease** | rs1217106 | -1.381 | 1.745 | 0.429 |
|  | rs12203592 | -6.773 | 3.072 | 0.027 |
|  | rs1318845 | -0.868 | 1.679 | 0.605 |
|  | rs6749467 | 0.660 | 1.741 | 0.704 |
|  | rs72808024 | 0.992 | 1.709 | 0.561 |
|  | rs7514956 | 4.784 | 1.670 | 0.004 |
|  | rs77205736 | -1.063 | 2.224 | 0.633 |
|  | rs77255816 | -0.204 | 3.454 | 0.953 |
|  | ALL | 0.267 | 0.993 | 0.788 |
| **Vitiligo** | rs1217106 | 2.294 | 10.874 | 0.833 |
|  | rs12203592 | -7.630 | 19.153 | 0.690 |
|  | rs1318845 | 9.695 | 10.499 | 0.356 |
|  | rs6749467 | 6.998 | 10.857 | 0.519 |
|  | rs72808024 | -9.102 | 10.647 | 0.393 |
|  | rs7514956 | -13.579 | 10.390 | 0.191 |
|  | rs77205736 | 10.976 | 14.263 | 0.442 |
|  | rs77255816 | -4.584 | 21.367 | 0.830 |
|  | ALL | -0.302 | 4.306 | 0.944 |

Ads: Autoimmune diseases; PM: Particulate matter; NOx: Nitrogen Oxides.

**Supplementary Table 6.** The leave-one-out analysis for air pollution on ADs (East Asian population)

| **PM2.5 on Ads** | **SNP** | **BETA** | | **SE** | ***P*-value** | |
| --- | --- | --- | --- | --- | --- | --- |
| **Systemic lupus erythematosus** | rs10983735 | -4.770 | 6.217 | | | 0.443 |
|  | rs12203592 | -16.592 | 11.116 | | | 0.136 |
|  | rs34623735 | 10.181 | 5.448 | | | 0.062 |
|  | rs7225402 | 9.670 | 5.190 | | | 0.062 |
|  | rs77205736 | 1.675 | 6.108 | | | 0.784 |
|  | ALL | 3.717 | 3.908 | | | 0.341 |
| **PM2.5-10 on Ads** |  |  |  | | |  |
| **Systemic lupus erythematosus** | rs11067476 | 0.063 | 0.235 | | | 0.790 |
|  | rs374130827 | -0.178 | 0.207 | | | 0.390 |
|  | rs7630570 | 0.260 | 0.215 | | | 0.226 |
|  | rs80151214 | 0.123 | 0.212 | | | 0.561 |
|  | ALL | 0.063 | 0.108 | | | 0.562 |
| **PM10 on Ads** |  |  |  | | |  |
| **Systemic lupus erythematosus** | rs10097518 | -0.124 | 0.222 | | | 0.575 |
|  | rs17821828 | 0.175 | 0.220 | | | 0.427 |
|  | rs2424731 | 0.062 | 0.234 | | | 0.791 |
|  | rs62164084 | 0.046 | 0.069 | | | 0.500 |
|  | rs6503238 | -0.184 | 0.224 | | | 0.410 |
|  | ALL | 0.029 | 0.059 | | | 0.625 |
| **NOx on Ads** |  |  |  | | |  |
| **Systemic lupus erythematosus** | rs11821352 | 0.314 | 0.213 | | | 0.139 |
|  | rs183491 | 0.136 | 0.208 | | | 0.513 |
|  | rs4333856 | -0.340 | 0.214 | | | 0.113 |
|  | ALL | 0.040 | 0.194 | | | 0.835 |
| **PM2.5 on Ads** |  |  |  | | |  |
| **Rheumatoid arthritis** | rs11067476 | -0.142 | 0.248 | | | 0.567 |
|  | rs374130827 | 0.257 | 0.157 | | | 0.103 |
|  | rs7630570 | 0.218 | 0.170 | | | 0.198 |
|  | rs80151214 | 0.102 | 0.160 | | | 0.523 |
|  | ALL | 0.150 | 0.088 | | | 0.086 |
| **PM2.5-10 on Ads** |  |  |  | | |  |
| **Rheumatoid arthritis** | rs12594133 | -0.052 | 0.163 | | | 0.748 |
|  | rs2319367 | 0.326 | 0.149 | | | 0.029 |
|  | ALL | 0.154 | 0.188 | | | 0.414 |
| **PM10 on Ads** |  |  |  | | |  |
| **Rheumatoid arthritis** | rs10097518 | -0.235 | 0.175 | | | 0.178 |
|  | rs17821828 | 0.004 | 0.181 | | | 0.980 |
|  | rs2424731 | 0.364 | 0.174 | | | 0.037 |
|  | rs62164084 | -0.124 | 0.060 | | | 0.038 |
|  | rs6503238 | 0.243 | 0.177 | | | 0.169 |
|  | ALL | -0.055 | 0.082 | | | 0.503 |
| **NOx on Ads** |  |  |  | | |  |
| **Rheumatoid arthritis** | rs11821352 | -0.121 | 0.168 | | | 0.471 |
|  | rs183491 | -0.146 | 0.162 | | | 0.370 |
|  | rs201266522 | 0.014 | 0.167 | | | 0.934 |
|  | rs4333856 | 0.140 | 0.180 | | | 0.438 |
|  | ALL | -0.036 | 0.085 | | | 0.671 |

Ads: Autoimmune diseases; PM: Particulate matter; NOx: Nitrogen Oxides.
